# A bibliometric analysis of alternative drug therapy options in the treatment of androgenetic alopecia

**DOI:** 10.1111/jocd.16427

**Published:** 2024-06-14

**Authors:** Aditya K. Gupta, Daniel Taylor, Shruthi Polla Ravi, Tong Wang, Mesbah Talukder

**Affiliations:** ^1^ Mediprobe Research Inc. London Ontario Canada; ^2^ Division of Dermatology, Department of Medicine, Temerty Faculty of Medicine University of Toronto Toronto Ontario Canada; ^3^ School of Pharmacy BRAC University Dhaka Bangladesh

**Keywords:** androgenetic alopecia, bibliometric analysis, low‐dose oral minoxidil, sublingual minoxidil, topical finasteride

## Abstract

**Background:**

Oral finasteride and topical minoxidil formulations are the only FDA‐approved drug therapies for androgenetic alopecia (AGA). Research into dutasteride, topical finasteride, and nontopical minoxidil (low‐dose oral and sublingual) formulations in the treatment of AGA has spiked within recent years. Early findings show that these alternative drug therapies may have similar to improved efficacy and safety profiles relative to the conventional treatment options.

**Aims:**

Conducting a bibliometric analysis, compare trends in publications on these alternative drug therapies, identify key contributors, evaluate major findings from top‐cited articles, and elucidate gaps in evidence.

**Methods:**

A search was conducted on the Web of Science database for publications on the use of alternative drug therapies in the treatment of AGA. A total of 95 publications, published between January 2003—March 2024, and their citation metadata were included in the analysis.

**Results:**

Dutasteride showed the greatest (*n* = 37) and longest (20+ years) history of publications, as well as the highest cumulative citations (*n* = 914); however, nontopical minoxidil showed a burst in research activity within the last 5 years (*n* = 33 publications since 2019). A relatively low number of randomized control trials (*n* = 3) for nontopical minoxidil suggests a need for higher‐quality evidence.

**Conclusions:**

Our analysis reveals major trends, contributors, and gaps in evidence for alternative drug therapies for AGA, which can help inform researchers on their future projects in this growing field of study. There is enthusiasm for exploring off‐label formulations: nontopical forms of minoxidil (oral and sublingual), topical finasteride, and mesotherapy.

## 1 INTRODUCTION

Androgenetic alopecia (AGA) is the most common nonscarring alopecia driven by complex genetic predisposition and follicular sensitivity to androgens.^1^, ^2^ The disease affects up to 50% of males (male androgenetic alopecia, MAA) and females (female pattern hair loss, FPHL), and can have profound psychological impacts on patients.^3^, ^4^ AGA is hallmarked by hair follicle miniaturization, which is believed to be mediated by microinflammation of the follicular bulge, abnormal sensitivity of the hair follicle to circulating androgens, irregularities in arrector pili muscles; most likely, some combination of these factors.^5^, ^6^

The two FDA‐approved drug therapies for male AGA are oral finasteride and topical minoxidil. For female AGA, only topical minoxidil is FDA‐approved. Finasteride acts as an inhibitor of 5α reductase type II to reduce the conversion of testosterone to dihydrotestosterone, which has high potency for the androgen receptor (AR).^7^ The mechanism by which minoxidil counteracts AGA is less clear, but it has been shown to stimulate protective Wnt/β‐catenin signaling, suppress inflammatory mediators, induce vasodilation near the hair follicle, and reduce expression of 5α reductase type II.^8^

Whilst oral finasteride and topical minoxidil are established and effective drug therapeutics in the treatment of male AGA for over 25 years, neither of these agents is necessarily the best treatment option for all patients due to unwanted side effects and sometimes less than optimal efficacy. Namely, oral finasteride use can be associated with undesired sexual dysfunction, particularly in males, while topical minoxidil has been shown to cause irritant contact dermatitis.^7^, ^9^ As such, alternative drug therapies for AGA are an attractive avenue for patients.

In recent years, dutasteride, topical finasteride, and nontopical minoxidil (including low‐dose oral and sublingual) formulations have been studied to evaluate their relative efficacy and safety profiles compared to the conventional AGA drug therapies.^10^, ^11^ Specifically, dutasteride, a more potent inhibitor of 5α reductase type II, as well as being a type I inhibitor, has been shown to significantly increase total hair count with a similar safety profile compared to oral finasteride.^12^ Meanwhile, topical finasteride and nontopical minoxidil formulations have demonstrated efficacy, as well, these different routes of administration that may be more acceptable to some patients.^13^, ^14^

A bibliometric analysis of the literature on these alternative drug therapies for AGA will allow insight into the state and progression of research in this rapidly expanding field.^15^ Through the identification of key contributors and research trends, we can determine which types of studies are under‐represented and bridge the gaps between inter‐collaborating groups to help guide future research efforts.

## 2 MATERIALS AND METHODS

Using the Web of Science (WoS) database, we conducted a literature search on 22 March 2024 with the following search formulas: dutasteride ([Title] TI = [dutasteride] AND ALL = [androgenetic alopecia]), topical finasteride (TI = [topical finasteride] AND ALL = [androgenetic alopecia]), and nontopical minoxidil (TI = [oral minoxidil] OR TI = [sublingual minoxidil] AND ALL = [androgenetic alopecia]). No date restrictions were used to filter the results. Non‐English articles, conference proceedings, and studies that did not study AGA or report the drug formulation were excluded. Full metadata eligible records, including authorship, affiliated institutions, citations and cited sources, were exported to Microsoft Excel. VOS Viewer (ver.1.6.20) and Excel were used for analysis and visualization.

## 3 RESULTS

### 3.1 Publication trend analysis

According to our literature search (Figure 1), there were a total of 95 publications on alternative drug therapies for the treatment of AGA, with the earliest publication in 2003. Figure 2 shows annual output of publications by year for each alternative drug therapy with cumulative citations, according to the WoS database. Dutasteride shows the greatest history of publications (20+ years), the greatest number of publications (*n* = 37), the highest in total citations (*n* = 914) and H‐index (17) among the three alternative AGA drug therapies, indicating a long‐standing interest in this treatment option. However, low‐dose oral and sublingual minoxidil formulations have received a spike of research interest within the last 5 years, with a total of *n* = 36 publications and *n* = 619 citations (H‐index = 15).

**FIGURE 1:**
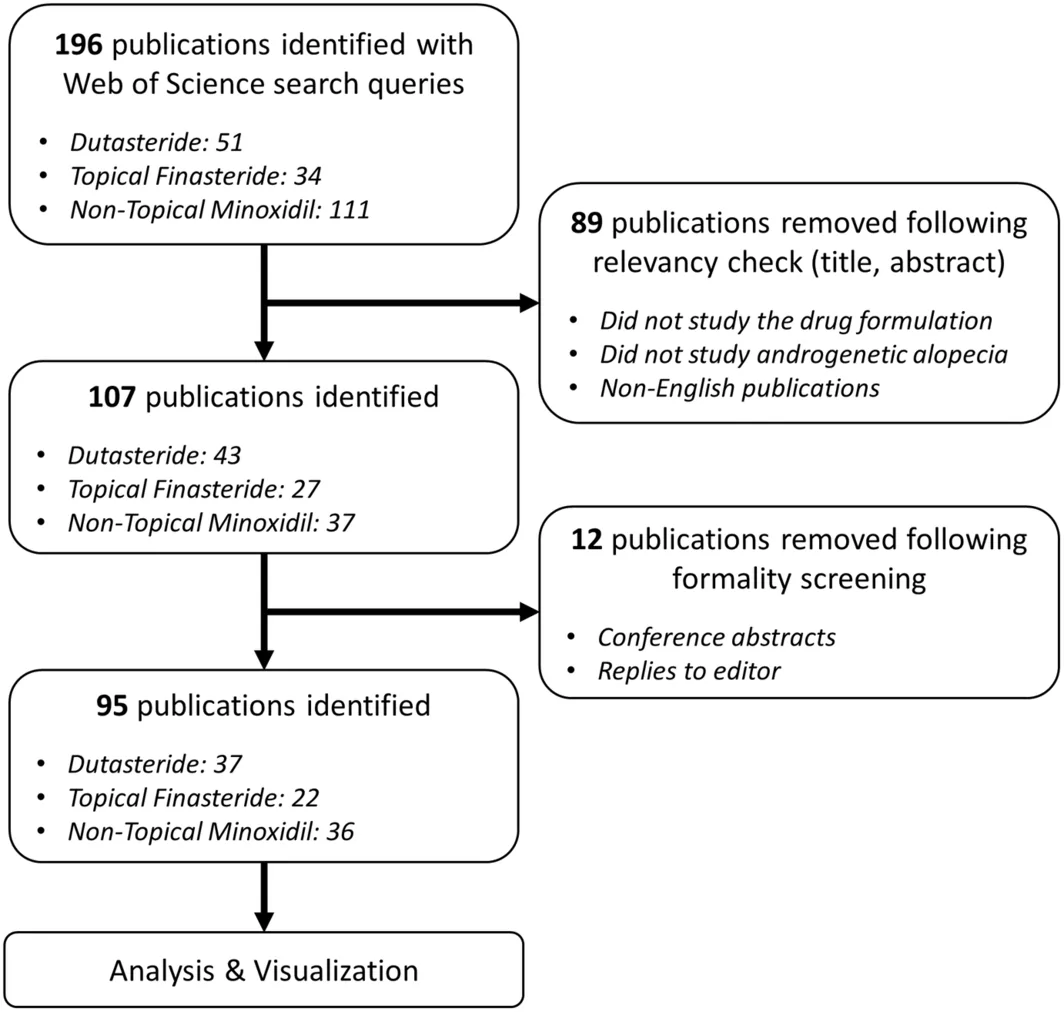
Publication screening flowchart.

**FIGURE 2:**
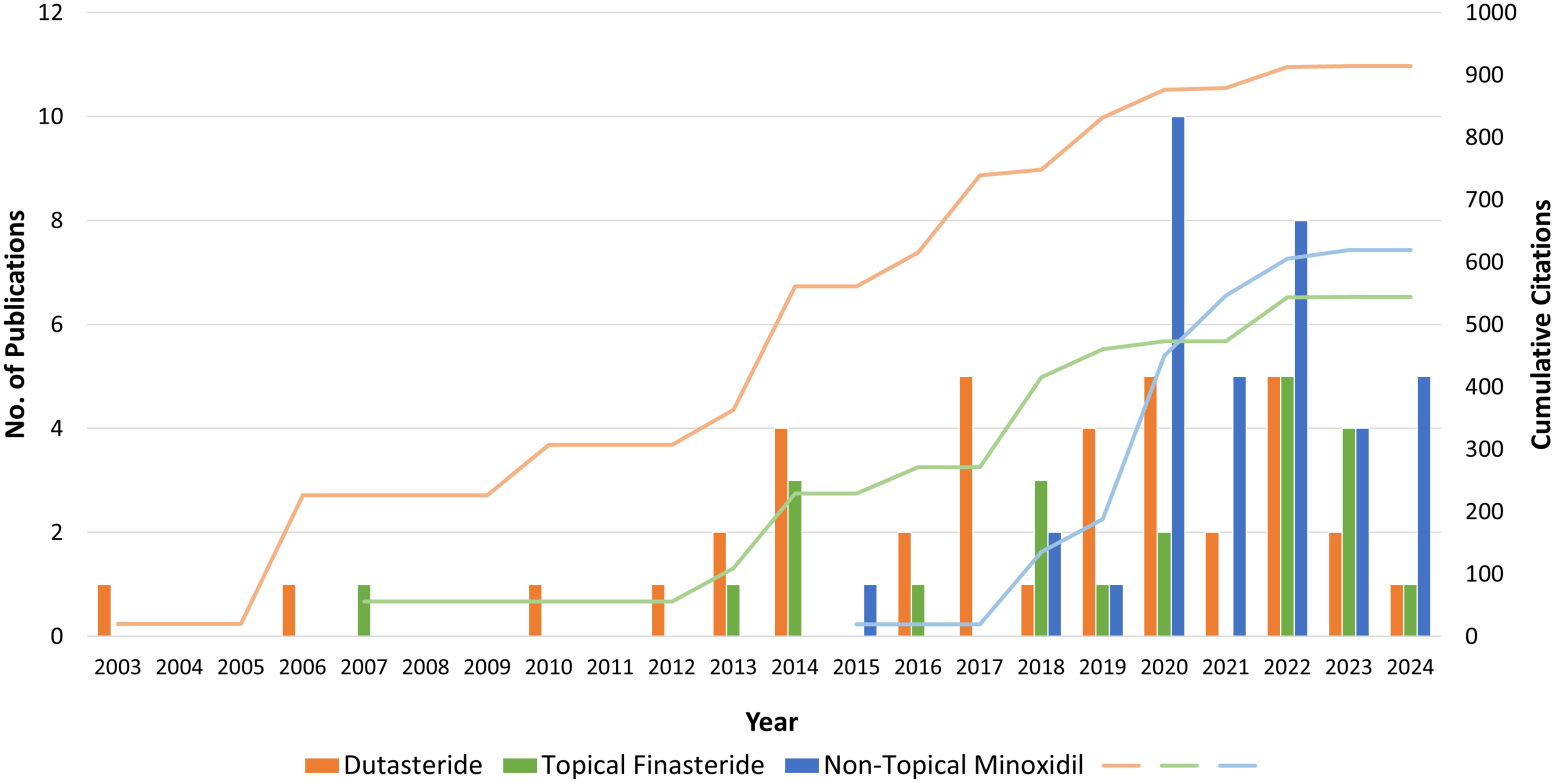
Annual research activity (publications and cumulative citations) of alternative drug therapies for AGA. AGA, androgenetic alopecia.

The full list of publications has been further categorized by publication type (Figure 3). Topical finasteride shows the highest density of randomized control trials (8 publications, 36% of total), whereas nontopical minoxidil exhibits the lowest density of both randomized control trials (3 publications, 8% of total) and meta‐analyses/systematic reviews (3 publications, 8% of total).

**FIGURE 3:**
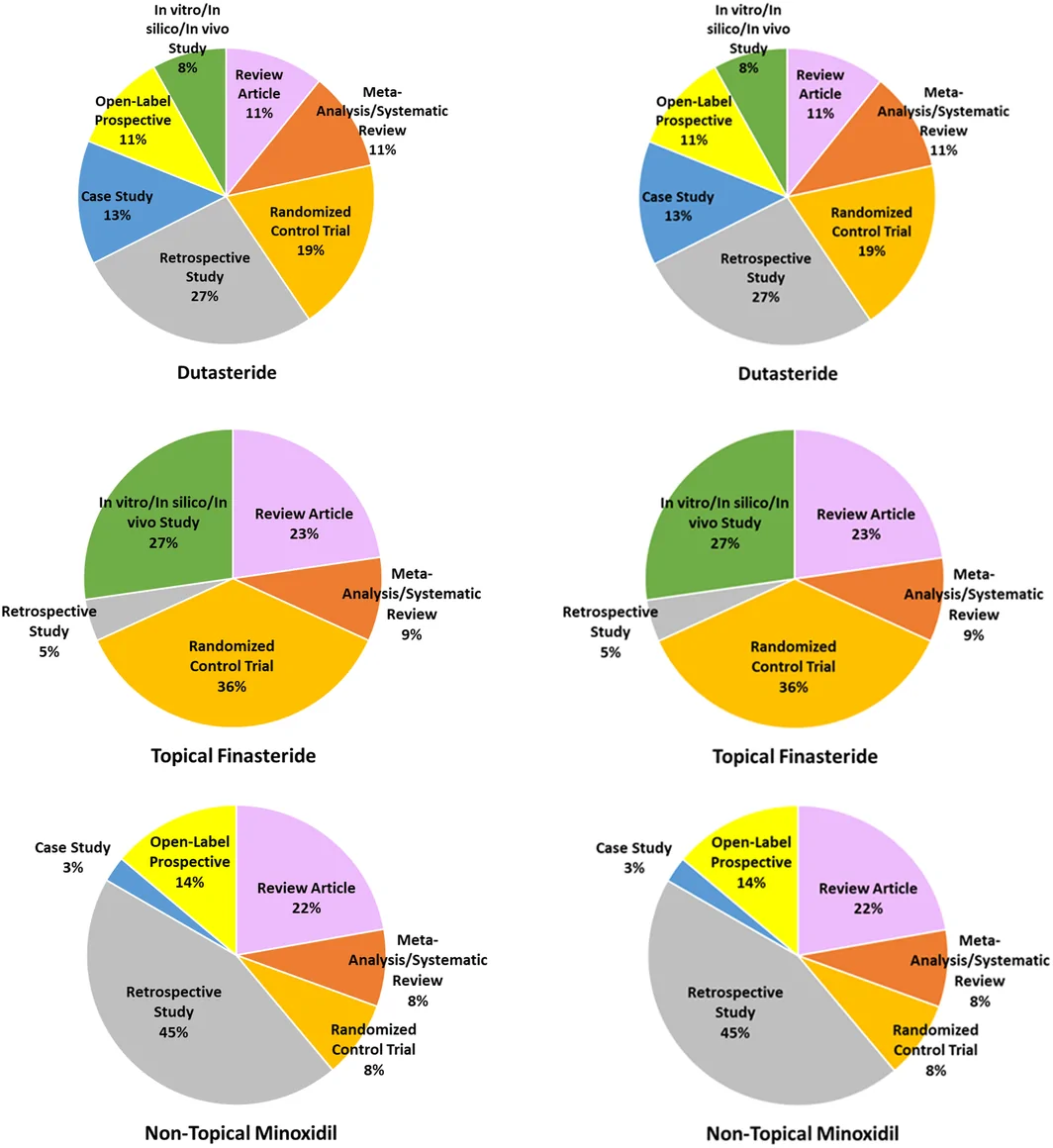
Publication type distribution for each alternative drug therapy for AGA. AGA, androgenetic alopecia.

### 3.2 Authorship and regional analysis

A total of 393 authors, 169 institutions, and 29 countries contributed to the body of research on alternative drug therapies for the treatment of AGA. With respect to total publications, the most prolific contributors were Sergio Vano‐Galvan (*n* = 8), Oscar Moreno‐Arrones and David Saceda‐Corralo (*n* = 7 each), whereas the most cited authors (with two or more publications) were Rodney Sinclair (*n* = 158), Won‐Soo Lee (*n* = 134), Makoto Kawashima and Ryoji Tsuboi (*n* = 115 each) (Table 1).

**TABLE 1:** Top‐contributing authors and affiliated institutions for publications on alternative drug therapies in the treatment of AGA, ranked by total publications and citations. Citation comparison limited to authors that have contributed two or more publications to the field of study.

Rank	Author: Publications	Affiliated Institution	Count (%)
1	Vano‐Galvan, S.	University of Alcala	8 (8.4%)
2	Morena‐Arrones, O.	University of Alcala	7 (7.4%)
3	Saceda‐Corralo, D.	University of Alcala	7 (7.4%)
4	Gupta, A.	University of Toronto	6 (6.3%)
5	Sinclair, R.	University of Melbourne	6 (6.3%)

Rank	Author: Citations	Affiliated Institution	Count (%)
1	Sinclair, R.	University of Melbourne	158 (7.6%)
2	Lee, W.	Yonsei University	134 (6.5%)
3	Kawashima, M.	Tokyo Women's Medical University	115 (5.5%)
4	Tsuboi, R.	Tokyo Women's Medical University	115 (5.5%)
5	Juhasz, M.	University of California	98 (4.7%)

Abbreviation: AGA, androgenetic alopecia.

Country coauthorship analysis shows collaboration patterns between different research groups around the world, where connection strength is determined by repeated collaborations. This analysis revealed five major clusters of collaboration for publications concerning alternative drug therapies for AGA; one centered on the USA, another focused around Spain, Brazil, and Australia, one centered around South Korea and India, another centered on Canada and Bangladesh, and a final cluster that involved Italy and Switzerland (Figure 4A). By visualizing average publication year, we see that recent publications in the field of study have been driven by collaborative efforts between Spain and Brazil (Figure 4B).

**FIGURE 4:**
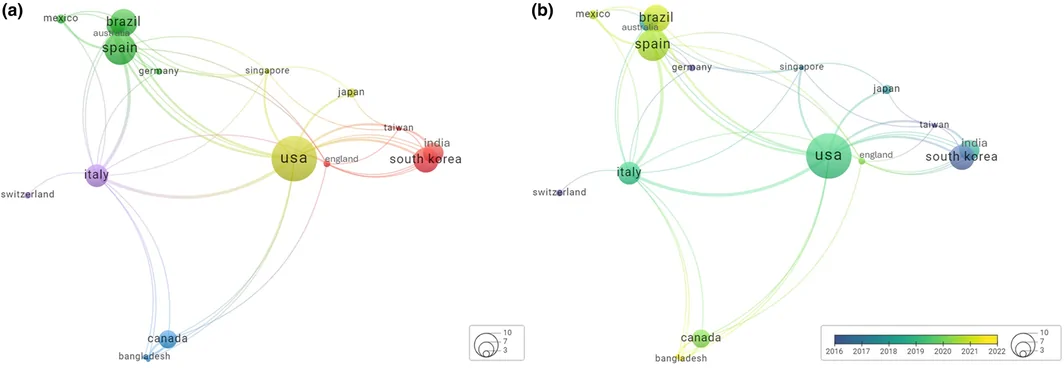
Country coauthorship analysis visualization. Analysis was limited to countries that authored two or more publications within the field of study (*n* = 18). Relative size of label and circle represents number of publications, and line thickness represents frequency of collaboration. (A) Clustering visualization. Different colors represent clustering according to coauthorship network analysis. (B) Average publication year. Color represents average year of publication from each country.

Author cocitation analysis reveals groupings of authors that are often cited together on publications, revealing clusters that may represent shared research themes. Our cocitation analysis reveals three major clusters, representing the three alternative drug therapies included in our literature search, and highlights the authors that bridge the gaps between these research interests (Figure 5).

**FIGURE 5:**
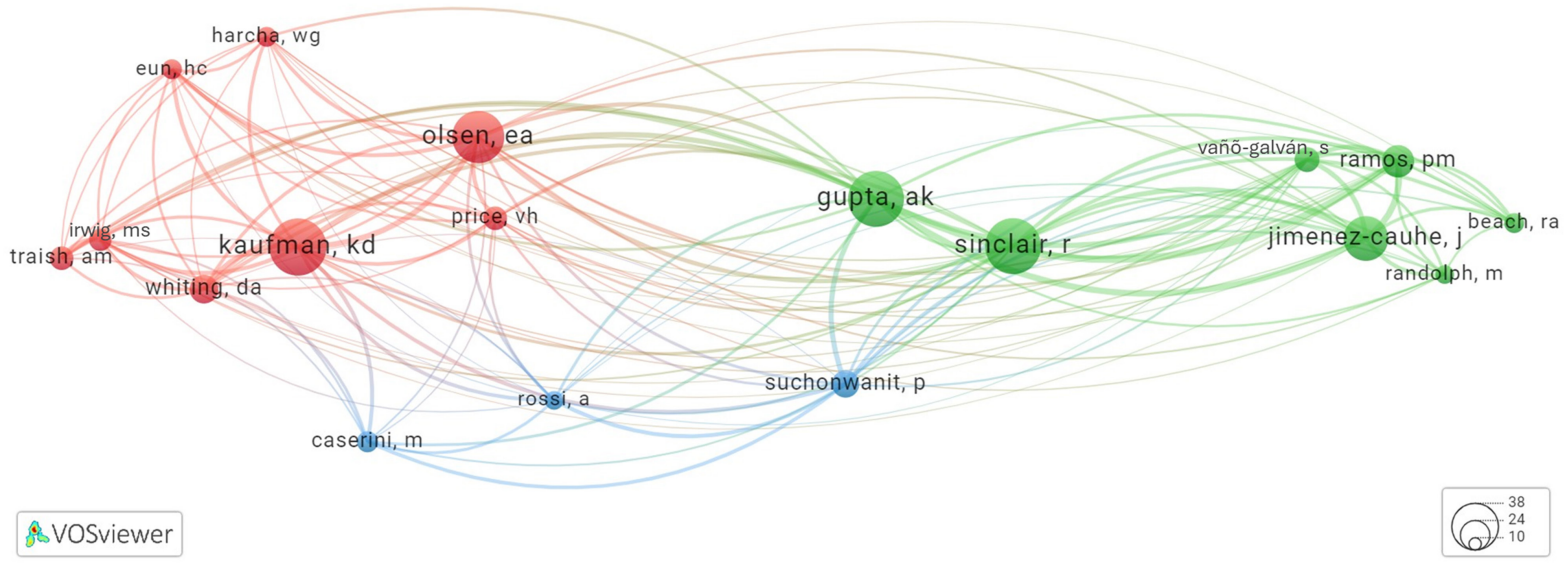
Author cocitation analysis visualization. Analysis was limited to first authors that were cited more than 15 times for publications within the field of study (*n* = 18). Relative size of label and circle represents number of citations, and line thickness represents frequency of cocitation between authors. Colors represent clustering according to cocitation network analysis.

### 3.3 Analysis of highly‐cited papers

By analyzing top‐cited publications, we can get an impression of the dermatology research community's knowledge and perception of these alternative drug therapies in the treatment of AGA. The top‐cited papers for each alternative drug therapies are listed in Table 2.

**TABLE 2:** Top‐cited publications for each AGA alternative drug therapy.

AGA Alternative Drug Therapy	Citation Ranking	Publication	Citation Count	Altmetric Score^a^
Dutasteride	1.	Olsen et al.,^17^ The importance of dual 5α‐reductase inhibition in the treatment of male pattern hair loss: Results of a randomized placebo‐controlled study of dutasteride versus finasteride	206	31
	2.	Harcha et al.,^16^ A randomized, active‐ and placebo‐controlled study of the efficacy and safety of different doses of dutasteride versus placebo and finasteride in the treatment of male subjects with androgenetic alopecia	84	13
	3.	Eun et al.,^18^ Efficacy, safety, and tolerability of dutasteride 0.5 mg once daily in male patients with male pattern hair loss: a randomized, double‐blind, placebo‐controlled, phase III study	81	15
Topical Finasteride	1.	Lee et al.,^19^ A Systematic Review of Topical Finasteride in the Treatment of Androgenetic Alopecia in Men and Women	70	40
	2.	Kumar et al.,^20^ Development of liposomal systems of finasteride for topical applications: design, characterization, and in vitro evaluation	56	0
	3.	Suchonwanit et al.,^21^ A randomized, double‐blind controlled study of the efficacy and safety of topical solution of 0.25% finasteride admixed with 3% minoxidil versus 3% minoxidil solution in the treatment of male androgenetic alopecia	55	92
Nontopical Minoxidil	1.	Sinclair,^22^ Female pattern hair loss: a pilot study investigating combination therapy with low‐dose oral minoxidil and spironolactone	83	338
	2.	Ramos et al.,^23^ Minoxidil 1 mg oral versus minoxidil 5% topical solution for the treatment of female‐pattern hair loss: A randomized clinical trial	62	43
	3.	Jimenez‐Cauhe et al.,^24^ Effectiveness and safety of low‐dose oral minoxidil in male androgenetic alopecia	53	54

Abbreviation: AGA, androgenetic alopecia.

^a^ Altmetric Attention Scores are based on online interactions with publication, including news outlets and social media platforms.

The three most highly‐cited publications for dutasteride use in the treatment of AGA include three randomized placebo‐controlled trials investigating the efficacy and safety of dutasteride when compared to finasteride and placebo,^16^, ^17^ or placebo alone.^18^ These studies all found dutasteride to be superior in improving hair regrowth compared to both finasteride and placebo, with a similar safety profile to finasteride.

The three highly‐cited papers for topical finasteride in the treatment of AGA include a systematic review of a set of randomized control trials,^19^ an early pharmacokinetics study on the finasteride topical delivery method,^20^ and a randomized control trial comparing finasteride admixed in a topical minoxidil solution to topical minoxidil alone.^21^ The systematic review concludes that topical finasteride is safe and effective in the reduction of hair loss; however, the evidence is limited despite promising preliminary results.^19^

Finally, the three highly‐cited publications for nontopical applications of minoxidil include an open‐label prospective study with combination low‐dose oral minoxidil and spironolactone,^22^ a randomized control trial comparing low‐dose oral against topical minoxidil,^23^ and a retrospective study investigating the use of low‐dose oral minoxidil in male AGA patients.^24^ These studies conclude that therapies using low‐dose oral minoxidil may be comparably safe and effective in reducing hair loss in male and female AGA patients to traditional drug therapies, but note that larger controlled trials are necessary to conclude these results and optimize dosage.

## 4 DISCUSSION

Alternative drug treatments for AGA provide patients with options with more potent therapeutic effects or manageable side effect profiles, and, as such, are deserving of research attention. The results of the present bibliometric analysis demonstrate that, though the majority of studies on these alternative drug therapies are recent, there are small and varied collections of literature for each of these treatment options that show promising results. The burst in publications and citations within the past 10 years suggest that health care providers and researchers are interested in evaluating and promoting evidence‐based findings of these alternative drugs and formulations into clinical practice.

Dutasteride has the longest history of publication and is perhaps the best candidate to be adopted into standard care among the alternative treatment options, with an extensive list of randomized control trials (RCTs, 7 total studies), long considered to be the gold standard of evidence, and a fair collection of meta‐analyses (4 total) that report on the success of dutasteride in these trials. However, the majority of these RCTs occurred before 2018, suggesting that the push for large‐scale RCTs investigating dutasteride is lessening.

Topical finasteride holds the smallest literature portfolio, with only 22 total publications, but is supported by a great proportion of RCTs (8 total studies) that report equivalent levels of efficacy to oral finasteride formulations while minimizing systemic side effects. However, there is currently no standardized formulation of topical finasteride that has been tested across these RCTs. A current meta‐analysis that evaluates RCTs from within the last 2 years may provide even greater evidence for topical finasteride as a bonafide treatment option for AGA.

Low‐dose oral and sublingual minoxidil formulations show the greatest jump in research interest over the last 10 years. However, nontopical minoxidil is lacking in evidence from RCTs (3 total studies) compared to the two other alternative drug therapies, with most of its evidence being derived from retrospective studies (16 studies, 44% of all studies) and open‐label prospective studies (5 studies, 14% of all studies). A recent RCT from Asilian et al.^14^ showed that low‐dose oral minoxidil had similar efficacy and safety profiles compared to the first‐line topical formulation, suggesting it may be a good alternative drug therapy option for certain patients, but further large‐scale trials should be carried out to support these results.

Coauthorship and cocitation analyses reveal a high degree of collaboration on impactful publications that, more often than not, crosses international borders. These outcomes suggest that the approach to research on these alternative drug therapies considers a diverse set of perspectives and patient populations, which is a healthy foundation as research on these therapeutic options continues into the future.

### 4.1 Limitations

WoS was chosen for our search and metadata collection because it has the greatest literature coverage among the top academic research databases, including SCOPUS and PubMed. However, we recognize that any one database cannot perfectly encompass all available literature and interaction data. Additionally, while VOSviewer assists in the visualization of bibliometric data, it may not capture all relevant publications within the networks the program generates.

## 5 CONCLUSION

Alternative drug therapies in the treatment of AGA have received a burst of research activity within recent years. While preliminary results are promising, there are some recognizable deficits in the evidence that can be elucidated through bibliometric analysis. Dutasteride has the greatest history of publication and citation among the alternative drug therapy options. The off‐label formulations, nontopical minoxidil (oral, sublingual, and mesotherapy) and finasteride (topical and mesotherapy) have received the most research activity within the last 5 years; however, lack high‐quality evidence due to a paucity of randomized control trials. Researchers can use these findings to make better‐informed decisions with their future projects to have an optimal impact on the body of research.

## AUTHOR CONTRIBUTIONS

Conceptualization, AKG, MT; Data Curation, DT; Formal Analysis, DT; Investigation, DT; Methodology, DT, MT; Project Administration, AKG; Resources, AKG; Supervision, AKG; Validation, SPR, TW; Visualization, DT; Writing – Original Draft Preparation, DT; Writing – Review and Editing, AKG, SPT, TW, MT.

## FUNDING INFORMATION

No funding was received for conducting this study.

## CONFLICT OF INTEREST STATEMENT

Aditya K. Gupta, Daniel Taylor, Shruthi Polla Ravi, Tong Wang and Mesbah Talukder have no competing interests to declare that are relevant to the content of this article.

## ETHICS STATEMENT

Authors declare human ethics approval was not needed for this study.

## Data Availability

The data that support the findings of this study are available from the corresponding author upon reasonable request.
